# Notes and Formulæ

**Published:** 1889-02

**Authors:** 


### PRACTICAL NOTES AND FORMULÆ.

**Laxative in Child-bed.**—Dr. M. H. Lee of Tenn., recommends the following:

℞ Ol. Ricini., . . . . . . . . ℥j

Syr. Rhei. Aromatic . . . . . . ʒv

Alcoholcum . . . . . . . . ʒiv

Ess. Pip. . . . . . . . . gtts v. to x.

M. Sig. Tablespoonful every hour until relieved. In extreme cases 2 to 4 tablespoonfuls every hour or two until relieved. (Shake the bottle before taking.

**Prairie Itch.**—In treating prairie itch, I have not had a failure in three hundred cases, using the following combination ; from there to ten applications completing the cure ;

℞ Hydrarg. chlor. mitis . . . . . gr. 160
Sulphuris sublimat . . . . . . dr. iv
Vaselini . . . . . . . . . oz. ij

M Sig.—Apply to affected surface at bedtime. Wash the skin with soap and water every other night.

DR. W. T. JOHNSON,
In *Med. World.*

**Eczema of the Head**—First wash the head of the child with soap and water and then apply the following ointment:

℞ Acid. salicylic, . . . . . . grs. xxv

Tinct. benzoin, . . . . . . ʒj

Vaseline, . . . . . . . ℥j

M. —*Med. World.*

**Incontinence of Urine.**—A great many females are unable to hold the water when laughing, coughing, or making any violent exertion. The best remedy for this condition is the following combination :

℞ Fl. ext. ergotæ, . . . . . . . ℥i.

Tinct. belladonnæ, . . . . . . ʒijss

Tr. nux vomicæ, . . . . . . ʒiij.

Aquæ, qs. ad., . . . . . . . ℥iij.

M. Sig: Teaspoonful three or four times a day.— *ed. Waif.*

**Earache.**—A liniment is recommended by Paresi for this affection, composed of—

℞ Camphorated chloral, . . . . . parts v
Glycerine, . . . . . . . . " xxx
Oil of sweet almonds, . . . . . " x

It is applied twice daily on soft cotton, being introduced as far as possible into the ear, and may also be rubbed behind the ear. The pain is almost instantly relieved, and the inflammation in many cases is subdued. The Liniment must be kept in carefully closed bottles.
—*Med. World.*

**Palpitation.**—℞ Tr. aconiti rad. gtt. xxjv., Aquæ qs. ad, ℥ iij. M. Sig: Teaspoonful three times a day in water. In some instances fluid extract of veratrum viride, in drop doses three or four times a day, acts very promptly and beneficially.—*Prof. Alonzo Clark.*

**Treatment of Warts by Arsenic.**—Pullin (quoted in *Philadelphia Med. News*, Feb. 18, 1888) cured warts occurring under the finger-nails, in the case of a child four years of age, by giving one minim of Fowler's solution (thrice daily?), for ten days. He says he has cured numbers of other cases in the same way.—*Satellite.*

**Treatment of Warts.**—Where creosote-salicylic plaster cannot be had, Unna recommends the following:

| | | |
|---|---|---|
| ℞ | Acid salicylic, | ʒ iij. |
| | Creasoti, | ʒ vj. |
| | Ceræ, | |
| | Adapis, | ää q. s. |

Make a firm ointment which will adhere to the skin.

**Emulsions in the Treatment of Skin Diseases.**—Dr. Valentine Knaggs used the following in a case of "eczema-lupus":

| | | |
|---|---|---|
| ℞ | Bismuth subnitrat., | ʒ j. |
| | Vaselin, | ℥ j. |

Fiat ung.

Place the ointment in a warmed mortar; incorporate with it one hundred and sixty grains gum acacia. Then add two drachms of hot water, mixed with sixteen grains of boric acid.

In psoriasis, Knaggs used the following:

| | | |
|---|---|---|
| ℞ | Paraffin molle, | ℥ ss. |
| | Pulv. zinci oxidi, | ʒ j. |
| | Acidi carbolici, | gr. j.—M. |

Fiat ung.

Mix with it, in the manner described above, eighty grains gum acacia, eight grains boric. acid, one drachm glycerine, and one-half to an ounce of water.—*Prov. ed. Jour.*, March 1, 1888.

**Cough Remedy**—Dr. L. M. Nichols writes:—For the distressing cough of Phthisis, I have found the following prescription most excellent.

| | | |
|---|---|---|
| ℞ | Creosote, | m viii |
| | Bis. Subnitrate | ʒ j |
| | Cherry Laural water, | ℥ j |
| | Morphia Sul. | gr. iss. |

M. S. Shake, give a teaspoonful in water, every 8 hours until cough is checked, and quietude restored.

I have had several cases of Phthisis with the most persistent coughs and the above prescription has never failed to give the very much needed rest in every case.

